# Prognostic factors in sudden sensorineural hearing loss: a retrospective study using interaction effects

**DOI:** 10.5935/1808-8694.20130083

**Published:** 2015-10-08

**Authors:** Chin-Saeng Cho, Young-Jin Choi

**Affiliations:** a Professor (Department of Otorhinolaryngology-Head & Neck Surgery, College of Medicine, Eulji University).; b Associate Professor (Department of Healthcare Management, Eulji University).; Department of Healthcare Management, College of Healthcare Industry, Eulji University.

**Keywords:** hearing loss, sudden, regression analysis, vertigo

## Abstract

The prognostic significance of vertigo in patients with idiopathic sudden sensorineural hearing loss (SSNHL) remains a matter of debate.

**Objective:**

This paper aims to verify the difference between a group with vertigo and a group without vertigo, and to analyze vertigo's validation as a prognostic factor in patients with SSNHL.

**Method:**

This study involved 183 patients with SSNHL. A *t*-test was used to compare group A (SSNHL with vertigo, n = 31) and group B (SSNHL without vertigo, n = 152). Also we want to verify the interaction effects between vertigo and other prognostic factors using multiple regression analysis.

**Results:**

There was a significant difference between group A and group B: the initial hearing level of group A was lower than group B, and their treatment onset was also shorter. In addition, vertigo itself didn't affect hearing improvement, but the interaction variable between vertigo and initial hearing level did affect hearing improvement significantly.

**Conclusion:**

The clinical characteristics of patients with vertigo did not directly affect hearing improvement with SSNHL; however, vertigo had an influence on SSNHL though its interaction with the initial hearing levels.

## INTRODUCTION

Sudden sensorineural hearing loss (SSNHL) is a loss that is greater than 30 dB in three contiguous frequencies and that occurs in less than 3 days^1^. SSNHL affects approximately 5-20 people per population of 100,000. It is almost always unilateral and is commonly associated with tinnitus and aural fullness. Multiple treatment protocols and agents have been proposed to treat SSNHL. Steroids, antiviral agents, anticoagulants, vasodilators, and others have been proposed as therapeutic agents to treat SSNHL^2^, ^3^, ^4^.

A maximum of 32% to 65% of cases of SSNHL may recover spontaneously. Prognosis for recovery is dependent on a number of factors, including patient age, presence of vertigo at onset, degree of hearing loss, audiometric configuration, and time between the onset of hearing loss and treatment. Even though some studies have reported vertigo as a prognostic factor to SSNHL, it is often considered a poor prognostic factor and the effect of vertigo as related to SSNHL^5^, ^6^, ^7^ is still debated. The reason for these inconsistent results is that vertigo is not a specific disease, but rather a symptom caused by many different etiologies. Although there were some attempts to verify the relationship between the results of caloric tests and prognoses^8^, ^9^, ^10^, the relation between SSNHL and vertigo has not been clearly determined.

## METHOD

This study used the medical records of 183 patients with SSNHL. All patients experienced idiopathic unilateral sensorineural hearing loss that developed within 3 days and excluded other known pathologies, including Meniere's disease, autoimmune disease, ototoxicity, or neoplasm.

The patients had a minimum hearing loss of 30 dB at three consecutive frequencies. Patients received steroid treatment (injection of prednisolone 60 mg/kg for six days then tapered over four days) started concomitantly with low molecular weight dextran, and checked 3 months after treatment. This study was approved by the institution's Ethics Committee and given permit number EU12-31.

The hearing loss classification was as follows: mild (26~40 dB), moderate (41~55 dB), moderately severe (56~70 dB), severe (71~90 dB), and profound (over than 91 dB). The Siegel^11^ classification was used to evaluate the hearing improvement of patients on the last visit, using an average gain in four audiometric speech frequencies of 500 Hz, 1,000 Hz, 2,000 Hz, and 4,000 Hz.

Patient characteristics and clinical details are given in Table 1. In 183 patients (108 female and 75 male) with a mean age of 45.11 (± 15.79) years, 152 patients did not have vertigo. The degree of hearing loss was relatively evenly distributed: mild (15.8%), moderate (18.0%), moderately severe (17.5%), severe (23.0%), and profound (25.7%). Treatment onset was distributed: within 3 days (161 patients), and over 3 days (22 patients).

**Table 1:** Demographics and clinical characteristics of the study population.

Factors	No. (%)
Age	

≤ 10	3 (1.6)
11-20	6 (3.3)
21-30	26 (14.2)
31-40	29 (15.8)
41-50	45 (24.6)
51-60	34 (18.6)
≥ 61	40 (21.9)

Vertigo	

(+)	31 (16.9)
(-)	152 (83.1)

Treatment onset (days)	

≤ 3	161 (88.0)
4-7	15 (8.2)
8-10	3 (1.6)
11-28	3 (1.6)
≥ 29	1 (0.5)

Initial hearing level	

Mild	29 (15.8)
Moderate	33 (18.0)
Moderately severe	32 (17.5)
Severe	42 (23.0)
Profound	47 (25.7)

## RESULTS

Statistical analyses were carried out with PASW Statistics ver. 18.0. A *t*-test was performed to identify statistically significant differences between group A (SSNHL with vertigo, n = 31) and group B (SSNHL without vertigo, n = 152). Multiple regression analysis was used to find prognostic factors associated with hearing improvement, and analyze the interaction effects of vertigo.

**Figure 1:**
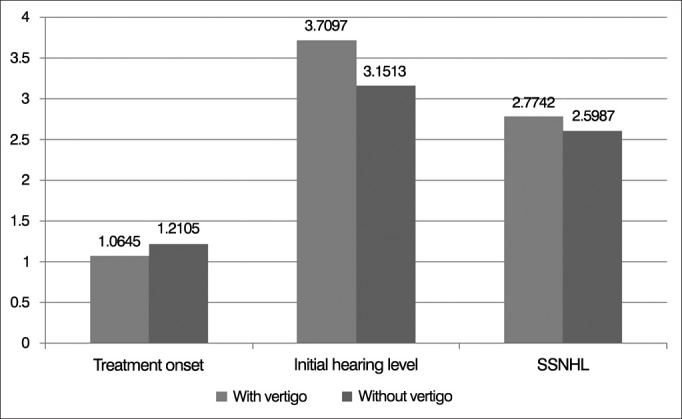
The difference between with vertigo and without vertigo.

The *t*-test results revealed that no significant difference in SSNHL between the two groups existed; however, a significant difference of 0.05 was found in the initial hearing level and treatment onset, as shown in Table 2 and Figure 1.

**Table 2:** Results of comparison between with vertigo and without vertigo.

Vertigo		Means	S.D.	*p*-value
Treatment onset	With	1.0645	0.24973	.035**
	Without	1.2105	0.63696	

Initial hearing level	With	3.7097	1.65718	.046**
	Without	3.1513	1.35568	

SSNHL	With	2.7742	1.30919	.501
	Without	2.5987	1.32355	

*p* < 0.01.

* *p* < 0.1;

** *p* < 0.05;

In addition, multiple regression analysis was used to identify predictors of hearing improvement in patient with SSNHL. Treatment onset, initial hearing level, and vertigo were used as independent variables while hearing improvement based on Siegel's classification was used as dependent variables. In the regression analysis results in Table 3, vertigo was insignificant as a prognostic factor of hearing improvement. Both the treatment onset and the initial hearing level were significant at 0.01. Also, the regression model was accepted at 0.01 (F = 7.973) and these factors explained 24.2% of the variance in SSNHL.

**Table 3:** Results of regression analysis with vertigo, initial hearing level, and treatment onset.

Variables	β	F	*p*-value
Vertigo	.007	.036	.850
Initial hearing level (IHL)	.176	8.600	.000***
Treatment onset (TO)	.466	58.946	.000***

*p* < 0.1;

*p* < 0.05;

*** *p* < 0.01.

We analyzed the interaction effects between vertigo and initial hearing (or treatment onset). After including interaction variables (Vertigo x TO, Vertigo x ITL), we investigated the following equations using multiple regression analysis.

$$\text{Y = α}+{\text{β}}_{1}{\text{ Vertigo + β}}_{2}{\text{ TO + β}}_{3}\;\left(\text{Vertigo x TO}\right)\text{ + ε: Equation 1}$$

$$\text{Y = α}+{\text{β}}_{1}{\text{ Vertigo + β}}_{2}{\text{ ITL + β}}_{3}\;\left(\text{Vertigo x ITL}\right)\text{ + ε: Equation 2}$$

As shown in the results in Table 4, the interaction effect between vertigo and treatment onset was invalid. But the interaction effect between vertigo and initial hearing level was valid at 0.01. The validation was checked as follows: β was identified as significant at 0.01, and the R^2^ of model 2 (0.250) was larger than the R^2^ of model 1 (0.213).

**Table 4:** Analysis of interaction effects between vertigo and treatment onset, initial hearing level.

Models	Variables	B	F	R^2^	ΔR^2^	F
	Vertigo (A)	.055	.762			
1	Treatment onset (TO)	.242	19.103***	.059	-	3.755**

	Vertigo (A)	.110	.697			
2	Treatment onset (TO)	.089	.092	.059	.0	2.229**
	Vertigo x Treatment onset (TO)	.169	.420			

Models	Variables	B	F	R^2^	ΔR^2^	F

	Vertigo (A)	.016	.195			
1	Initial hearing level (IHL)	.463	61.174***	.213		9.578***

	Vertigo (A)	.467	10.484***			
2	Initial hearing level (IHL)	1.617	32.810***	.250	.037	6.410***
	Vertigo x Initial hearing level (IHL)	-.1258	17.200***			

*p* < 0.1;

** *p* < 0.05;

*** *p* < 0.01.

## DISCUSSION

This research focused on the prognostic factors associated with SSNHL. Initial hearing level, treatment onset and vertigo were investigated as prognostic factors. We also analyzed the interaction effects of vertigo, which was not verified clearly, as a prognostic factor of former research.

Firstly, following the comparison results between group A (SSNHL with vertigo, n = 31) and group B (SSNHL without vertigo, n = 152), initial hearing level and treatment onset were significantly different between the two groups at the 0.05 level, but SSNHL was insignificant. This result supports Ahn et al.^12^ in proving a significant difference between the two groups.

Secondly, from the results of the multiple regression analysis, initial hearing level and treatment onset were significant to hearing improvement while vertigo was insignificant. In the former researches, the initial hearing level negatively affected SSNHL^13^, or the initial hearing level did not affect SSNHL except in patients with profound cases^14^. Siegel^11^ showed that there was no significant relation between the initial hearing level and SSNHL. Our findings added to the research stream of Kwon et al.^14^ and Byl^13^.

Siegel^11^ showed that there was no significant relation between treatment onset and SSNHL while other researchers found that treatment onset affected SSNHL^14^, ^15^. Our research supported the findings that treatment onset affected SSNHL significantly (*p* < 0.01). Especially, there were debates over whether vertigo was a prognostic factor toward hearing recovery rate. Sheehy^15^ presented that the recovery rate of a patient with vertigo was lower than the recovery rate of a patient in the non-vertigo group. Vertigo decreased the hearing improvement rate in the patients with profound cases^16^. Also, Simmons^17^ hypothesized that vertigo with SSNHL may be the result of a membrane break near the vestibule. Khetarpal^18^ studied patients using SSNHL and vertigo, and suggested that vertigo may be caused by biochemical alterations in the inner ear. Kiris et al.^19^ presented that between the group with vertigo and the group without vertigo there was no difference statistically. Our research results also supported Kiris et al.^19^.

Finally, we carried out research to verify the interaction effects between vertigo and other independent variables (initial hearing level, treatment onset). The research results showed that vertigo did not affect SSNHL directly, but we found interaction effects between vertigo and initial hearing level. This result supported former studies^15^, ^16^, and clearly showed that vertigo affected hearing improvement through interaction with the initial hearing level.

This research has a value to verify that the treatment onset and the initial hearing level affect hearing improvement. More important contribution is to find the interaction effects of vertigo and initial hearing level to predict hearing improvement with SSNHL patients. That is, it supports former research studies^17^, ^18^ only presented that vertigo was a significant factor to SSNHL through case studies or comparison methods. But, this research generalized the vertigo effect of hearing improvement with SSNHL statistically using interaction effects method. These results will need to be verified through future studies.

## CONCLUSION

This research analyzed prognostic factors associated with hearing improvement with SSNHL. A *t*-test was used to compare between group A (SSNHL with vertigo, n = 31) and group B (SSNHL without vertigo, n = 152) with data from 183 patients, and multiple regression analysis was conducted. The results showed that vertigo did not directly affect SSNHL. However, there is considerable evidence of interaction effects between the initial hearing level and vertigo.
